# Connections for Matters of the Heart: Network Medicine in Cardiovascular Diseases

**DOI:** 10.3389/fcvm.2022.873582

**Published:** 2022-05-19

**Authors:** Abhijeet Rajendra Sonawane, Elena Aikawa, Masanori Aikawa

**Affiliations:** ^1^Center for Interdisciplinary Cardiovascular Sciences, Division of Cardiovascular Medicine, Department of Medicine, Brigham and Women’s Hospital, Harvard Medical School, Boston, MA, United States; ^2^Center for Excellence in Vascular Biology, Division of Cardiovascular Medicine, Department of Medicine, Brigham and Women’s Hospital, Harvard Medical School, Boston, MA, United States

**Keywords:** network medicine, cardiovascular disease, systems biology, protein–protein interaction (PPI), gene regulatory network (GRN), coexpression network

## Abstract

Cardiovascular diseases (CVD) are diverse disorders affecting the heart and vasculature in millions of people worldwide. Like other fields, CVD research has benefitted from the deluge of multiomics biomedical data. Current CVD research focuses on disease etiologies and mechanisms, identifying disease biomarkers, developing appropriate therapies and drugs, and stratifying patients into correct disease endotypes. Systems biology offers an alternative to traditional reductionist approaches and provides impetus for a comprehensive outlook toward diseases. As a focus area, network medicine specifically aids the translational aspect of *in silico* research. This review discusses the approach of network medicine and its application to CVD research.

## Introduction

Cardiovascular disease (CVD) is a leading cause of mortality and morbidity worldwide (1). In 2018, the World Health Organization reported that CVD was responsible for 31% of global deaths (17.9 million deaths each year) (2). In the United States, CVD prevalence was 49% (126.9 million adults) in 2018 (3). CVD comprises a number of conditions involving the heart and vasculature, including coronary artery disease (CAD), myocardial infarction (MI), heart valve disease, aneurysm, peripheral artery disease (PAD), heart failure (HF), cardiac arrhythmia, cardiomyopathy, stroke, and pericarditis (4). Intermediate risk factors that contribute to CVD development and progression include hypertension, dyslipidemia, diabetes, obesity, sleep apnea, and hyperuricemia with serious consequences on the heart and vasculature (5). The risk factors affecting such conditions are modifiable behavioral factors (e.g., smoking, high cholesterol diet, high-salt diet, and physical inactivity) combined with non-modifiable predispositions (e.g., age, race, ethnicity, sex, and genetics). Other major contributors to the burden of CVD include pollution (6) and other environmental factors (7, 8). Although the long-standing view is that males are at higher risk for CVD, similar lifetime risk is found in both sexes (9). However, females have additional risk factors, such as emotional stress and depression, menopause, pregnancy complications, family history of CVDs, and inflammatory disease, that increase their overall risk of CVD (10).

## Current Challenges in Cardiovascular Disease Research

### Shared Pathobiology Among Different Cardiovascular Diseases

**FIGURE 1:**
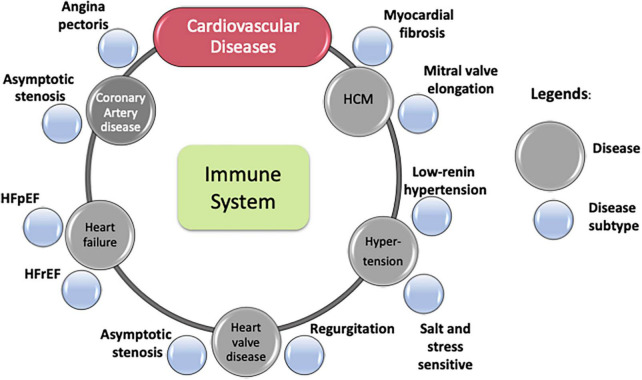
Cardiovascular diseases and some of their subtypes. Immune process lies at the core of most cardiovascular diseases.

While phenotypically diverse, many CVDs share pathophysiologic intermediaries. For example, atherosclerosis is a chronic inflammatory condition characterized by plaque buildup in the intima of blood vessels. This dominant cause is shared by various vascular diseases through mechanisms, such as (i) thickening of arterial walls in CAD and PAD, (ii) plaque rupture and thrombosis in its onset of acute complications (e.g., MI), and (iii) changes to aortic media causing aneurysms. However, components of the immune system underlie atherosclerosis development and aggravation. Dysregulation of innate immune systems may accelerate atherosclerosis through mechanisms such as impaired efferocytosis, sustained macrophage activation, and activation of the NLRP3 inflammasome (11, 12). Similarly, adaptive immune mechanisms may promote atherosclerosis through interferon-γ, tumor necrosis factor-α, and interleukin-17 (IL-17) (13, 14). While inflammation appears to promote several atherosclerotic vascular diseases, including CAD, accumulating evidence suggests various CVDs share inflammation as a common pathology (Figure 1). Such shared pathologies motivate a systems-based, holistic view of CVD research, along with independent studies of different diseases.

### Heterogeneity Within the Same Disease

Despite shared pathologies, individual CVDs that affect different organs and/or tissues naturally display unique sets of symptoms and outcomes. Within each CVD, increasing evidence suggests heterogeneity in clinical manifestations and underlying mechanisms. Diverse clinical presentations of CAD, including asymptotic stenosis due to atheroma, angina pectoris due to thrombosis, and ST-segment elevation MI (STEMI) vs. non-STEMI (15), are some examples of phenotypic heterogeneity in disease expression. Heterogeneity in hypertrophic cardiomyopathy (HCM) in patients with diverse sarcomere-independent morphological features (16) is another example.

Disease subtyping or phenotyping, as per nosology, derives from clinical presentations or other observable characteristics without suggesting underlying mechanisms. It is done by clustering subjects with similar observable clinical characteristics. Such phenotypic assessments (i.e., identifying disease attributes that describe clinical differences in presentation of diseases) helps refine diagnoses and determine treatment strategy. Akin to diverse clinical presentation of the same disease in different patients, heterogeneity also manifests in patients’ drug response, such as seen in HF with either preserved (HFpEF) or reduced (HFrEF) ejection fraction. Traditionally viewed as different stages of the same disease, evidence now indicates that these conditions may be different disorders, with different etiologies and responses to therapy. While they share many common risk factors (e.g., obesity, hypertension, and diabetes) (17), many drug trials failed to demonstrate a similar efficacy in HFpEF to what can be achieved in HFrEF. This discrepancy highlights the importance of considering pathophysiology, not just clinical presentation, in disease classification and the subsequent design of clinical trials (18).

However, disease endotyping is defined by distinct functions and pathobiological mechanisms as well as altered molecular pathways. Endotyping improves patient stratification, leading to more accurate diagnoses and tailored therapeutic strategies. Proper endotyping requires a framework that can integrate pathways and mechanisms with phenotypic features, molecular measurements, and demographic data (19–21).

### Multifactorial Nature of Cardiovascular Diseases

Only a few CVDs are monogenic, including hypercholesterolemia, either familial (mutant gene: LDLR) or autosomal recessive (mutant gene: ARH); sitosterolemia (mutant genes: ABCG5/8) (22); and single mutation disorders such as Marfan syndrome, Loeys-Dietz syndrome, vascular Ehlers-Danlos syndrome, to name but a few (23). The pathogenesis of most CVDs is multifactorial and involves many complex genetical risk factors, including multiple pathogenic genes, hundreds of single nucleotide polymorphisms (SNPs), copy number variations, and genetic loci (24). Moreover, the combinatorial effect of multiple SNPs imparting a strong heritable component of CVDs indicates the presence of multiple overlapping pathological mechanisms that remain unidentified. In the context of CVDs (25, 26), genome-wide association studies (GWAS) indicate that identifying missing heritability may require understanding and functional characterization of risk factors and other intermediate traits leading to disease development and progression (27).

Cardiovascular diseases involve not only genetic, but also various environmental and lifestyle factors. These causal factors determine individuals’ CAD risk and cause multilayer heterogeneity in CVD patients. Along with genetics, data from other omics modalities (e.g., transcriptomics, proteomics, metabolomics, and epigenetics) provide a way to examine the disruptions and dysregulation caused by disease. While each type of omics data provides helpful information about changes in molecular composition or chromatin states, integrating them into unified models can give valuable insights into mechanisms underpinning disease pathology (28). The success of precision medicine in CVDs requires (i) consideration of complex overlaps of phenotypes and shared pathologies between different CVDs; (ii) the presence of endotypes within the same disease; (iii) accounting for heritability through intermediate risk factors; and (iv) integration of different omics data. Such an approach should also be able to handle big biomedical data, incorporate interdisciplinary research methodologies, and provide a framework to integrate multidimensional information about the disease.

Network medicine provides a framework that considers different components and constituents in the system and integrates them in an architecture that unravels the disease etiology, facilitates drug target discovery, and enables proper disease endotyping (21, 29).

## Network Medicine: A Tool for Cardiovascular Disease Research

**FIGURE 2:**
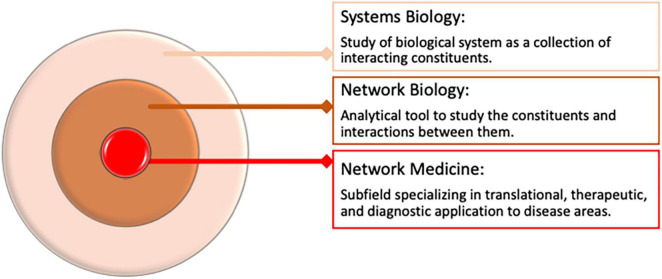
Overview of Network Medicine in Biomedical Data Analysis and its relation to systems biology.

Networks are analytical tools that allow us to represent complex associations between different entities from complex datasets. Networks may comprise either co-abundance, physical, or regulatory interactions between different biomolecules such as messenger ribonucleic acid (mRNA), proteins, or metabolites. For example, networks may comprise interactions between genes whose expression patterns are correlated or similar. In some cases, networks may represent abstract associations between entities such as genes shared between diseases and correlations shared between clinical features. Network medicine is a subfield of network biology, an area of systems biology that specializes in biomedical applications of network theory, from understanding etiology to discovering drugs and biomarkers (21, 29–31) (Figure 2).

The network medicine approach is often contrasted with the traditional reductionist, “magic-bullet” approach of finding unitary features (e.g., genes, SNPs, or drugs that influence the phenotype or disease), but each approach complements the other. Network medicine uses the complex interplay between disparate entities−molecular, biochemical, or cellular−and binds them together into consistent substructures, allowing distillation of relevant biology from the data and providing a glimpse of higher-order organization in the system under study. The major difference between the reductionist approach, which reduces the process into basic units, and the systems-based approach, which considers biological systems as a whole, is that the systems-based approach reveals emergent properties observable only when they are whole and not by their individual constituents (32). On the surface, the disparities between these approaches exist, but each approach is incomplete without the other: individual molecular measurement does not provide holistic context and systems-level models cannot be built or validated without painstaking biomedical experiments. By leveraging the large amount of biomedical data generated, network medicine has ushered in a new era in biomedicine (33).

Network medicine takes its concepts and terminologies from the mathematical field of graph theory and from complex networks where “nodes” are components such as genes and proteins; the interactions between them are “edges” (34). Such architecture assimilates information into different components and explains how they interact with each other within a system. Studying the network’s structural characteristics allows us to identify important features of its components. Probing network properties involves structural or topological characteristics like degree of the node (the number of its nearest neighbors), the degree distribution (probability distribution of these degrees over the entire network), clustering coefficients (measure of the extent to which nodes in a graph cluster together), shortest path lengths between the nodes, diameter (shortest distance between the two most distant nodes), and the presence of giant connected components (connected component with significant portion of nodes in the network). Translating these characteristics to meaningful biological insights is one of the most useful hallmarks of network medicine. Koutroli et al. discuss the basic terminology and graph theoretic concepts of networks, including general network properties, definitions of centralities, information about subnetworks and motifs, and various ways of network visualizations (35).

### Network Medicine for Multiomic Data Integration

**FIGURE 3:**
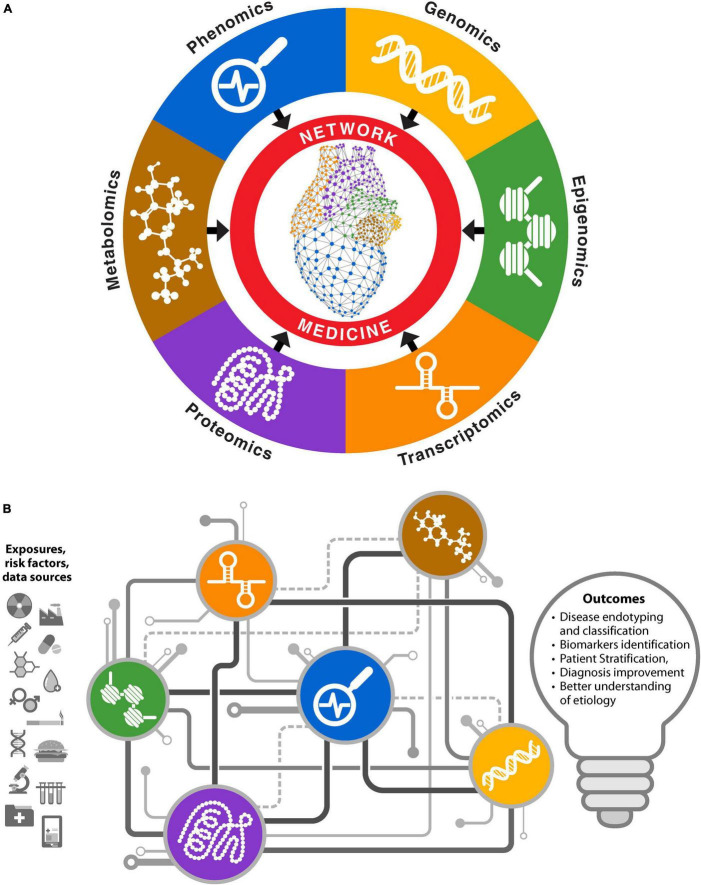
Network medicine approaches for multiomics biomedical data integrations. **(A)** Networks for individual omics types are constructed and analyzed separately. Information from each omics layer is aggregated to obtain biological insights. **(B)** Integrative approach where different omics data are used to infer interactions between various biomolecules. Different types of biomolecules from an omics platform are connected in coherent network structures, facilitating information exchange through mechanisms like message passing.

The advent of high throughput technologies, the decreasing cost of sequencing, and willingness in public to allow the use of biomedical data for research through biobanks and consortia has yielded a vast amount of data from different omics platforms as well as clinical and phenotypic information. Network medicine effectively integrates information from multiple omics data types and extracts meaningful information and mechanistic understanding from different omics layers (e.g., transcriptomics, proteomics, genetics, and phenomics) (Figure 3). Several challenges remain in leveraging these different omics datatypes, especially during integration (e.g., data harmonization, differences in scaling and normalization, matching genes to proteins and metabolites, and different batch effects in different omics layers). Krassowski et al. discuss major considerations during data integration (36). Joshi et al. provide a detailed discussion about each omics type in the context of CVD (37). Use of networks to analyze biomedical data from different omics platforms can be classified in two ways. One approach involves obtaining information from different omics layer separately and then assembling it. Because different biological components work together, information from each omics layers can provide a facet of the system under study. In this approach, separate networks are constructed for each omics layer using a variety of different algorithms, depending upon the omics type, to establish a relationship among the features. After analyzing the networks to identify important genes, proteins, or epigenetic marks relevant to the disease in each omics modality, the overlapping entities can be used to identify important biomolecules relevant to the underlying biology (Figure 3A). We can also identify relationships between different omics layers using this approach (38).

Another approach to network medicine-based multiomics integration involves simultaneously analyzing different components from different omics layers by incorporating them into unified models. Networks constructed with this approach may have different types of nodes and the nature of edges between different node types may differ. For example, genes, proteins, or epigenetic marks may be connected based on biological principals, signaling pathways, and central dogma, along with correlated abundance levels. The edges may be present among same or different node types such as protein–protein, protein–DNA, or mRNA–protein. Some network methods use mathematical inferences as an additional step to give weights to the interactions (39). This approach is akin to a complex electric circuit, where different components exchange information to construct a more coherent output that informs the system under study, in this case underlying biology of the system (Figure 3B). It also reflects the complexity of multiomics integration, where the function of a complete circuit cannot be inferred from its individual components. However, unlike an electric circuit, this approach does not need information from all omics sources; available omics data can be built into coherent models to probe the system. Examples of this approach include Passing Attributes between Networks for Data Assimilation (PANDA) (40), Similarity Network Fusion (SNF) (41), and PAthway Recognition Algorithm using Data Integration on Genomic Models (PARADIGM) (42), among others (39). The concept of multiplex or interconnected networks also falls within this category (43). Mishra et al. provide a detailed list of variety of methods and tools for integrative omics (44).

### Network Medicine and Artificial Intelligence

Network medicine, artificial intelligence (AI), and/or machine learning are at the core of systems biology, which aims to identify the molecular determinants of disease. Using various classifiers, machine learning—supervised or unsupervised—is a major tool for identifying data patterns. Advanced models of machine learning include deep learning models that allow construction of various architectures of neural networks and innovations in loss functions, consequently performing different tasks in a variety of applications. These learning-based approaches are valuable when identifying diagnostic, predictive, prognostic, or therapeutic signatures of disease, including biomarkers. Despite many parallels (e.g., unsupervised machine learning as modularity detection in networks), significant differences exist between networks- and learning-based approaches; primary among them is their black-box nature and lack of interpretability of the latter. In contrast to linear regression and decision trees, which are easier to understand and have fewer parameters, deep learning algorithms base their decision on intricate neural networks with a huge number of weight parameters. Even knowing all the weights does not allow comprehension of a model’s behavior, leading to its black-box nature.

One of the most lucrative outcomes of biomedical big data analysis is the use of multiomics approach to identify new molecular or clinical biomarkers of disease. Such biological signatures could include disease-associated proteins, mutations, deletions, and copy number variation of genes identified by various omics platforms. Biomarker discovery has benefitted greatly from the advent of AI, especially its ability to handle large multivariate and unstructured clinical data (e.g., electronic health records and data from imaging modalities such as chest computer tomography and X-rays). These types of data are not easily amenable for network medicine approaches due to their size, complexity, heterogeneity, incompleteness, and unstructured nature. However, the strength of network medicine lies in leveraging omics data to find important biomolecules that serve as “network biomarkers” (45). Such biomarkers are often identified using genes responsible for network topology changes due to disease. For example, biomarkers for major adverse cardiac events were identified using protein interactions and signaling pathways (46). Other advances include using network-based models to identify blood-based and circulating biomarkers (47–49). The following sections discuss some examples in which network medicine approaches identify CVD biomarkers. Similarly, AI-based biomarker discovery using deep phenotyping with multiomics and analysis of digital electrocardiogram and data from wearable devices shows promising results in HF (50), left ventricular systolic dysfunction (51, 52), and arrhythmia (53). Although reviewing specific applications of AI in cardiovascular contexts exceeds the scope of this review, several interesting review articles address this area (54–58). Giordano and colleagues discuss the efficacy and application of machine learning and AI in clinical decision making when developing personalized models of patient care (59).

### Article Outline

Network medicine has grown tremendously over the years, producing many successful applications across various disease areas and pathological landscapes. This growth spurred several excellent review articles and books that discuss its different aspects (30, 60–62). Notable among these is a series of articles by Loscalzo and colleagues and other torchbearers for network medicine (29, 63, 64) who have discussed different facets of network medicine approaches that focus on specific disease areas, including pulmonology (60), pathobiology (65), cardiology (66, 67), and coronavirus disease 2019 (68–70). These reviews discuss various concepts, applications, applicability to specific problems, practices, pitfalls, and above all, the promises of network medicine.

**FIGURE 4:**
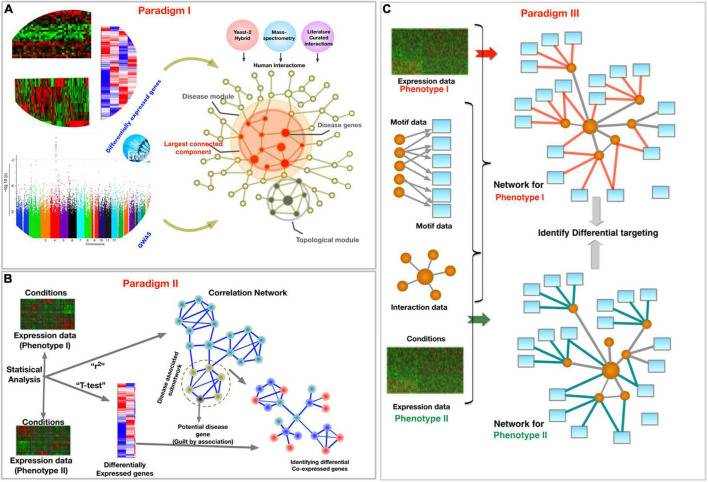
Schematic of three paradigms for combining biological networks with phenotype-specific biomedical data, such as a set of disease genes and transcriptomic profiles for case and control groups. **(A)** Identification of disease-associated network components within the interactome. **(B)** Co-expression-based network modeling to identify disease biomarkers. **(C)** Constructing phenotype-specific GRNs to identify perturbations and condition-specific regulatory changes. Figure borrowed from Sonawane et al. (71).

Many other interesting reviews and books discuss various aspects of network medicine in general and in the context of CVD. While some focused on discussing different omics technologies and how network methods can be leveraged to analyze them, others discussed individual network projects in detail or the philosophical underpinnings of the field, providing a context for understanding the contributions of network medicine to biomedical advances. The present review article is at the cross-roads of these ideas. We do not provide a chart of network methods, but rather try to inculcate an understanding that will empower researchers to select a specific network medicine approach, depending on data type, disease context, and outcome expectation. For this purpose, we rely on our previous review article (71), which discusses network medicine in the context of analyzing biomedical big data from different omics platforms. We also classified network medicine research into three different paradigms: (i) an interactome of protein–protein interactions (PPIs) with other omics data to elucidate disease context, (ii) pattern analysis in the co-abundance of measured analytes in disease, and (iii) deciphering gene regulation principles through phenotype-specific networks (Figure 4). Here, we use that classification template to discuss the application of network medicine and review several examples and applications in CVD contexts. While these paradigms provide a bird’s eye view of the field, we will cover CVD applications where PPIs and co-expression networks are used together.

## Network Medicine Paradigms for Cardiovascular Disease Research

### Applying Protein–Protein Interactions in Cardiovascular Diseases

Large-scale efforts to collect data have yielded far-reaching effects on biomedical research. For example, the Human Genome Project mapped the human genome and the Encyclopedia of DNA Elements (ENCODE) (72) provided functional elements of the genome. Consortia such as the Human Protein Atlas (HPA) and Genotype-Tissue Expression (GTEx) provided detailed atlases of human proteome and transcriptome in different tissues, respectively. Data from these efforts provided a comprehensive “parts-list” of cellular machinery that approaches like network medicine can assemble. Similarly, high-throughput mapping of protein—protein interactions (PPIs) is a significant milestone in biomedical research (72–77). PPIs are networks with proteins as nodes and interactions between them as connections. PPIs are a collection of interactions between proteins measured in various ways, including a yeast-two-hybrid system (78, 79), co-immunoprecipitation followed by mass spectrometry (80, 81), literature curation (82), or collection of various evidences (83–87). Primarily, PPIs are undirected because the direction of interactions between proteins is undefined. Previously, paradigm 1 of our earlier review article discussed different features of PPIs in detail (Figure 4A). Here, we recap some important features and mention the studies that have implemented them in the cardiovascular context. We do not intend to provide an exhaustive compendium of all the studies, but rather intend to discuss a few exemplar research studies to elucidate the efficacy of the approach.

#### Using Topological Properties of Nodes in Protein–Protein Interaction Networks

Protein–protein interactions can be used to study the importance of proteins via networks’ topological properties, such as “hubness” (i.e., nodes, or proteins, whose links with other nodes are higher than average) (88), and to identify densely interconnected subsets of proteins in the given biological context (89). Even though PPIs are incomplete (90, 91) and biased to contain edges of more extensively studied proteins, leveraging them helps identify important proteins in each disease context via the topological properties of individual nodes in the networks (92). Centrality measures like degree, closeness (i.e., the proximity a node to all other nodes), betweenness (i.e., the number of shortest paths traversing a node) allow study of the role a node (protein) plays in the information flow of the network (e.g., PPI) (93). The centrality properties of the nodes can be used to identify potential drug targets. For example, removing nodes may have a high impact on the stability of the disease network, thus indicating the lethality of the target. Certain combinations of centrality measures can elucidate the role of specific proteins in the PPI. Nodes with low degree and high betweenness indicate they are “connector-nodes” that bridge disjointed network neighborhoods, possibly representing different diseases, phenotypes, or pathways. Several examples of applying the network properties of PPIs to CVD exist (94). For example, NEDD9 critically connects adaptive and pathogenic fibrosis in the context of CVD found via its betweenness centrality (95). Sun et al. used centrality measures to compare various disease categories, providing novel ways to evaluate potential drug targets (96). Other applications of nodes’ topological properties to CVDs include heart failure (97), calcified aortic valve disease (CAVD) (98, 99), rheumatic heart valve disease (100), and ischemic stroke (101). Like network properties of individual genes, leveraging pathways’ properties can glean the hierarchical structure of disease organization. For example, control centrality identified the statistical significance of 66 pathways in type 2 diabetes (102). This approach also identified the peroxisome proliferator-activated receptor alpha (PPARα) pathway as a potential causal factor, and thus a drug target in vein graft disease (103).

When combined with phenotype-specific proteomics data, PPI networks also participate in identifying new molecular pathways or biomarkers of CVD. For example, extracting a cardiovascular-specific subnetwork along with mass spectrometry data enabled “knowledge-integrated biomarker discovery” in CVD (46). As discussed previously, the topological properties (e.g., centralities and modules) of nodes within a network can aid investigation of their biomarker status.

Integrating PPIs with gene expression levels, protein abundances, and genotype information may aid the study of disease heterogeneity and enable precision medicine in CVD. Combining patient-specific genotype and clinical data into PPI networks to obtain patient-specific genotype–phenotype relationships is critical for personalized medicine (66). For example, genetic variants can be mapped onto disease modules to create personalized networks, or “reticulotypes” (65, 68, 104). Maron et al. used the reticulotypes approach to construct an individualized network that distinguishes between hypertrophic and dilated cardiomyopathy (104). In respiratory diseases such as asthma (105), allergy (106), and chronic obstructive pulmonary disease (107), improved endotyping can be replicated in cardiovascular applications (108). Leopold et al. discussed endotyping in the context of CVD (109).

Proteins do not work in isolation; rather, they work together to perform different biological functions. In the PPI network, the presence of a high degree of clustering and topological modules (i.e., groups of proteins connected more among each other than with other randomly selected sets of proteins) reflects the formation of such protein complexes (29, 110). Often, such modules or clusters are specific to biological functions (111, 112). Various algorithms can identity modules and subnetworks. As discussed in depth by Lazareva et al., these methods use different strategies (e.g., greedy algorithms, evolutionary algorithms, diffusion-flows, or random walks), each with their own strengths and weakness (113).

#### Studying Disease Biology Using Protein–Protein Interaction Networks

Protein–protein interactions networks are undirected and, by design, lack disease context (114). In most cases, “seed” genes, identified through GWAS; differential abundance of analytes, such as proteins or genes; or various other means introduce disease context in the PPI network. The idea that each disease has a unique discrete subnetwork (115), and that such subnetworks overlap and share pathobiological mechanisms, has had a broad impact on the study of disease biology (116). The unified manner of viewing different diseases together has revolutionized the field (117).

Further, PPIs have been incorporated into a larger framework of multilevel networks containing interactions between genes, diseases, proteins, and drugs (118), and have been used to find associations between various CVDs (119). For example, some studies investigated GWAS-based seed genes in the context of subnetworks of PPIs in CAD (120–122). Ghiassian and colleagues performed one of the more interesting applications of disease biology on PPI in the context of CVD (117). Their study identified crosstalk between the molecular mechanisms underlying the inflammatory, thrombotic, and fibrotic processes that use endophenotype-specific modules as well as macrophages as key mediating cell types in these modules (117). A recent study used disease modules to investigate the pathobiology of the calcification process in the context of identifying several genes that overlapped with three other mechanisms (i.e., inflammasome, thrombosome, and fibrosome) (123). A different study examined disease subnetworks that use PPI in autophagy, a cellular process linked to hypertensive heart disease; the networks provided a global view of autophagy pathways, including candidate interacting proteins (124). PPI networks have allowed us to uncover pathways that drive abnormal development of the heart’s anatomical structures in congenital heart diseases (125, 126). In addition, PPI-based disease modules have been implemented in the context of calcific aortic valve disease (99). For example, we obtained proteomic subnetworks of calcific and fibrosa layers from calcified aortic valves and studied them in the context of metabolic, cardiovascular, inflammatory, and malignant diseases in humans using network closeness. This showed that the calcific stage subnetwork was closer to vasculitis, dermatomyositis, systemic lupus erythematosus, pulmonary embolism, pulmonary fibrosis, intracerebral hemorrhage, Takayasu arteritis, hypercholesteremia, bronchiolitis obliterans, non-alcoholic fatty liver disease, atherosclerosis, myocardial infarction, and hypercholesteremia. These important findings help overcome the incompleteness of PPIs, and allow for the study of diseases based on shared expression patterns and comorbidities. Another interesting aspect of the study was the leveraging of topological properties of nodes in the networks, as described above. The betweenness centrality of the calcific stage and the fibrosa layer stage network showed that fibronectin-1, along with PSMD3 and PSMA1, have high betweenness in both networks; all are known to contribute to CAVD (99). This study also provided a high-resolution spatiotemporal atlas of the combined valvular tissue, layer, and cell proteome as well as a comprehensive repository of the molecular drivers of CAVD.

#### Interactome and Drug Discovery

Protein–protein interactions network analysis also aids in the discovery of drug targets. Drug target discovery is a multifaceted and multicomponent process involving meticulous steps of progression target identification and prediction, followed by mechanistic validation studies that eventually lead to drug candidate design, screening, and clinical trials (127). Traditional methods of target or drug discovery are hypothesis-driven or biochemistry-informed. The brute-force methods of individually testing many drug candidates lead to high cost and have a high potential of failure. Reductionism in conventional drug discovery presents several challenges (68, 128). The systems-based approach differs from conventional means by efficiently integrating data and then studying the systemic effects of a drug by developing concepts like molecular disease networks (29). The strength of network medicine lies in its ability to integrate multiple sources of evidence and omics layers and identify potential targets of repurposed drugs, off-target effects, and mechanisms of action alongside empirical evidence needed for drug development (67, 129, 130). In contrast to the hypothesis-driven approach of drug discovery, which involves formulating a linear hypothesis and testing it with conventional means (e.g., genetically altered mouse strains), the systems-based approach utilizes unbiased omics, bioinformatics, and network analysis to predict clinical impact, ultimately accelerating the process and increasing the success rate. For example, our group studied inflammation in the context of chronic kidney disease, a major cardiovascular threat, to explore mechanisms by which indoxyl sulfate promotes macrophage activation (131). We demonstrated that unbiased proteomics analysis provided different clusters of proteins that shared an expression pattern over time. We studied the clusters using “network-closeness” to proteins of different diseases that use protein interaction networks to provide the disease context of the clusters. We showed that proteins in such clusters implicate significant associations with atherosclerosis and myocardial infarction, and that Notch signaling acts as a key regulator of pro-inflammatory macrophage activation by indoxyl sulfate, a uremic toxin. In the same study, *in vitro* mechanistic experiments further demonstrated that OAT/OATP-family transporters and the ubiquitin-proteasome pathway mediate indoxyl sulfate-induced Delta-like ligand 4-Notch signaling and macrophage activation (131).

Similarly, we used a systems-based approach to identify new regulators of macrophage activation through stimuli such as interferon-γ (IFN-γ) and IL-4. First, we identified proteins that increased and decreased in pro-inflammatory or in anti-inflammatory macrophages, respectively, and embedded them onto protein interaction networks. Next, we studied the closeness of these proteins to clusters of other diseases and determined that CAD was the closest disease cluster. Again, this approach derives credence from the aspect that disease genes do not act in isolation or randomly, but rather in consonance with similar biological pathologies. Subsequently, we used *in vitro* and *in vivo* validations, including single-cell analysis revealing heterogeneity, and established the role of ADP-ribosylation proteins PARP9 and PARP14 in human primary macrophages (127). The study also opened a new path toward global, unbiased assessment of ADP-ribosylated proteins in activated cells and tissues by using high-resolution mass spectrometry, which also involves network medicine for assessing biological significance (132, 133). Li and colleagues present another example of extensively applying network theory to drug discovery for CVD (119). They examined multilayer interactions between CVD drugs, targets, and genes to identify relationships between different cardiovascular disorders (119).

### Correlation-Based Network Approaches to Investigate Cardiovascular Disease Pathology

As discussed previously, phenotypic context does not come naturally to the interactome or PPI because they are a static map of protein interactions. Disease context must be embedded onto them through seed genes or proteins. By design, co-expression-based networks are built by using measured analytes in the disease or phenotypic context. In our previous review, paradigm 2 discussed different ways to leverage co-abundance information to construct co-expression networks that use a variety of network methods and correlation measures (Figure 4B). Identifying biomolecules expressed at similar levels can provide insight on their cooperative function or how they co-regulate each other or other constituents of the biological process (134–136). Among many methods that calculate co-expression networks, weighted gene correlation network analysis (WGCNA) remains among the most widely used methods in cardiovascular medicine. WGCNA builds co-expression networks by calculating the correlation between genes and organizes the network based on identified highly correlated clusters of genes. After identifying the “eigengene” (i.e., the first principal component of the expression matrix of the corresponding module) of each cluster as representative of that cluster, WGCNA determines its associations with disease or other clinical phenotypes, thus revealing which cluster is most informative of the given phenotype. Numerous applications of this method have been applied in a variety of CVD areas. For cardiac hypertrophy and failure, co-expression module analysis identified ZIC2 as a regulator of failing myocardium (137). Another study investigated cardiac hypertrophy by using co-expression analysis and micro-RNA (miRNA) data to identify the involvement of PPARα in sex differences (138). Regarding septic cardiomyopathy, WGCNA identified the critical role of various genes (139) in obesity-related cardiomyopathy (140), hypoplastic left heart syndrome (141), viral myocarditis (142), and ischemia-reperfusion in patients undergoing coronary artery bypass graft surgery (143). Some studies of pulmonary arterial hypertension imparted critical understanding of its pathogenesis by using gene co-expression networks (144–153). Analysis of transcriptomics networks in nearly 3,600 individuals with blood pressure measurement identified SH2B3 as a key regulator of blood pressure (154). In a major investigation, Bertero and colleagues used network analysis to implicate miRNA-130/301, which targets PPARγ in different cell types, in the development of pulmonary hypertension (155). In the context of hypertension, a comparison of co-expression hub genes and miRNA revealed important associations in the interaction between gene co-expression and miRNA (144). Others applied WGCNA analysis to diseases such as non-syndromic thoracic aortic aneurysm (156) and acute aortic dissection (157) using module trait association analysis. Genotyping and transcriptomics data from the MAGnet consortium helped Cordero and colleagues determine that PPP1R3A regulates HF pathology (158). Furthermore, a co-expression network investigation revealed central transcriptional regulators in macrophage activation under different stimuli, which is an important research problem in atherosclerosis development and progression (159).

#### Using Correlation Networks With Protein–Protein Interactions to Identify Key Driver Nodes

In addition to identifying disease modules and “eigengenes”, researchers frequently pair co-expression networks analysis with PPIs to identify modules’ molecular regulators. Key driver analysis (KDA) is innovative in this regard (160–162). KDA projects relevant disease modules (e.g., clusters from WGCNA) onto reconstructed Bayesian regulatory networks. The inference step of the Bayesian regulatory gene network (163) provides directionality to gene–gene interactions in co-expression modules that use text mining, which is then leveraged to determine the hierarchical order between genes, leading to identification of the most essential genes—the key driver genes (KDGs)—as master regulators of the modules under study (164, 165). SWItchMiner (SWiM) is another promising approach that integrates co-expression networks with PPIs (166). SWiM provides phenotype-relevant proteins that can act as therapeutic targets that function as a switch between different phenotypic states (e.g., proteins that can transition disease networks to control networks). Used to study ischemic and non-ischemic cardiomyopathy and other diseases, SWiM allowed construction of a new way of studying human disease networks that employ switch genes as molecular determinants of the diseases (167).

This article reviews some exemplary applications in CVD that use co-expression networks. Several studies investigated CAD using co-expression networks and KDGs extensively (168–170). For example, Zhao and colleagues devised an integrative approach to incorporate genotype information and transcriptomic profiles along with tissue-specific gene regulatory networks to identify potential key regulators of CAD (171). Further investigation of liver-specific gene regulatory networks revealed MAFF as an important key driver and transcription factor (TF) regulator of low-density lipoprotein receptors in CAD (172). Talukdar et al. used multi-tissue co-expression network modules and correlation between module eigengenes to identify KDGs in CAD (173). Large-scale data collection efforts focusing on CAD, such as the Stockholm Atherosclerosis Gene Network (STAGE) (174) and its continuation, Stockholm-Tartu Atherosclerosis Reverse Networks Engineering Task (STARNET), collected genetic and transcriptomic datasets from seven vascular and metabolic tissues (i.e., liver, subcutaneous adipose, visceral abdominal adipose, skeletal muscle, blood, atherosclerotic-lesion-free internal mammary artery, and atherosclerotic aortic artery) from 600 CVD patients. These studies accelerated “multi-tissue multiomics” integrative analysis (175–178). Comparative analysis of STAGE and STARNET co-expression networks showed broad reproducibility of results (179). Other important studies, such as the Integrated Personal Omics Profiling study (180) and the Pioneer 100 Wellness Project (181), accelerated CVD research in multiple directions. Leon-Mimila and colleagues provided a detailed review of various biomedical data collections in CVD, along with the omics information and integration results (28). Another study used similar approaches to investigate how antiretroviral therapy for HIV affects CAD (182).

Cardiovascular disease shares the same risk factors as cardiometabolic diseases including type 2 diabetes, chronic kidney disease, and non-alcoholic fatty liver disease. Hence, studying these diseases in the context of different tissues allows us to understand their common molecular framework. Notably, Cohain et al. used STARNET’s co-expression modules to investigate the conflicting roles of lipid and glucose metabolism in diabetes and CAD (183). While most co-expression analyses conclude by alluding to the roles of important genes in a module and their regulators in just one system, they replicated relevant multi-tissue, co-expression network modules (i.e., liver glucose and lipid determining) in three different studies, including an obesity cohort, GTEx, and a mouse model (183). Such multi-system confirmation adds due credence to downstream analysis and provides confidence for validated targets (183). Along with CVDs, the STARNET database is a useful resource to study cardiometabolic diseases. Koplev and colleagues used STARNET to construct co-expression-based gene networks between cardiometabolic diseases and CAD (184). Similarly, Shu et al. explored shared regulatory networks between type 2 diabetes and CVD and also identified 15 KDGs, including HMGCR, CAV1, IGF1, and PCOLCE, for both diseases (185).

Correlation network analysis is not restricted to transcriptomics data, and several studies show innovative application to other omics data (186, 187). For example, our group constructed co-abundance networks of proteins for IFN-γ and IL-4 stimulated in the human macrophage-like cell line THP-1, demonstrating that proteomics can identify GBP1 and WARS as potential regulators of pro-inflammatory signaling. Using a WGCNA framework, DNA methylation data elucidated associations between carotenoids and plasma lipid concentrations (187). Another study investigated modules in protein co-expression networks from human serum to find associations with various diseases like coronary heart disease, HF, and type 2 diabetes, and “eigenproteins” to predict future events and disease progression (188). An investigation of the topological properties of network proteins yielded new insights into hub properties, which were studied earlier primarily in protein–protein interaction networks but seldom in protein co-expression networks (189). However, applying WGCNA-like approaches to proteomics data requires consideration of the distribution, missingness, thresholding, and normalization for identifying relevant disease-specific modules (186).

### Leveraging Gene Regulatory Networks to Understand Cardiovascular Disease Pathology

Because co-expression networks themselves are symmetrical (i.e., do not include directionality between interactions), directionality pertaining to regulator and effector nodes require inclusion by design. Gene regulatory networks (GRN) comprise interactions between effector nodes, such as TFs, other signaling proteins, and receiver nodes that are genes (Figure 4C). While there is no consensus on the proper definition of GRN, most networks that include co-expression between genes use this broad terminology. Gene regulation studies primarily investigate the regulatory potential of TFs in cellular, tissue, or disease contexts. Many algorithms have been proposed to leverage different omics datatypes, primarily transcriptomics and transcription factor binding, to infer gene regulatory networks. To establish directionality between gene–gene interactions, suggested strategies include model-based approaches like Boolean networks, Bayesian network models; similarity-based approaches like linear and non-linear co-expression; and conditional mutual-information network models. Most apply to bulk data such as the Algorithm for the Reconstruction of Accurate Cellular Networks (ARACNE) (190) and context likelihood and relatedness (191), but some methods include single-cell data such as the SCENIC method (192), the GENIE3 algorithm, and the SCODE method (193). Methods, including PANDA (40), that explicitly model the role of transcription factor to infer their regulatory gene targets using message passing between co-expression and the interactome are useful across many diseases (194–197), sex-specificity (198), and the regulatory architecture of healthy human tissues (199). This message-passing approach now includes a variety of multiomics data types such as miRNA in PANDA using MicroRNA Associations (PUMA) (200), chromatin accessibility in Seeding PANDA Interactions to Derive Epigenetic Regulation (SPIDER) (201), and genotypes in Estimating the Genetic Regulatory Effect on TFs (EGRET) (202). In the context of CVD, an investigation of mitral valve disease that used PANDA to explore the role of serotonin 5-HT dysregulation on mitral valve interstitial cells revealed increased expression of 5-HTR2B and 5-HT receptor signaling (203).

Regulatory networks centered on transcription factors have played an important role in CVD research. Macrophage activation has an important implication in atherosclerosis. Network analysis of macrophage activation in response to bacterial lipopolysaccharide (LPS) revealed the role of activating transcription factor 3 and NRF-2 during inflammatory response (204). Using GRNs to integrate gene expression, histone modifications in promoters and enhancers, and TF motifs in different human tissues, Schmidt and colleagues showed that expression levels of relevant genes correlated with chromatin accessibility in different tissues (205). Another study used GRNs to explore the role of Toll-like receptors in transcriptional control of macrophage activation (206). A similar approach was considered for studying epigenetic architecture, histone modifications, and gene expression in pulmonary arterial hypertension (207). GRN analysis has aided the study of atherosclerotic plaques (208, 209) and heart failure (158). Moreover, differential network analyses are useful tools when describing the sources of dysregulation between two phenotypes (31, 210) (Figure 4C).

An emerging target of CVD therapeutics is non-coding RNAs, especially due to their stability in plasma and confinement in extracellular vesicles. Additionally, miRNAs and long non-coding RNAs (lncRNAs) serve as biomarkers of CVD (211–213). Particularly, lncRNAs regulate macrophage functions and thus play a significant role in CVD pathophysiology (214). Some studies determined a regulatory relationship between different mRNAs and non-coding RNAs (215, 216). Network medicine can investigate such an interplay of non-coding regulatory network mechanisms (217).

## Future Perspectives on Network Research in Cardiovascular Disease

Thus far, network-based studies in CVD have identified key molecular protagonists in disease progression. Many studies also identified changes to biological networks as sensors or mediators of the underlying pathobiology. Disease modules and their shared features between different diseases provide insight into a larger organization of molecular networks in different diseases, highlighting both unique and overlapping pathobiologies and instigating new ways to study diseases.

In certain areas, network medicine progress can aid CVD research, especially by resolving molecular mechanisms to infer proper endophenotypes. Like most complex diseases, CVD involves multiple common signaling and molecular pathways that are shared between different diseases. Combining these pathways can allow construction of unified disease networks. For example, one study performed endotyping in asthma by combining Ig-E, IL-5, IL-4, and IL-17 pathways (106). Similar networks can be constructed for specific CVDs (e.g., CAD or calcific aortic valve disease) by combining relevant pathways and signaling cascades. Importantly, unified networks are amenable dynamical simulations using Boolean logic transitions, where environmental stimuli or genotype risk can be incorporated as inputs and activation of specific pathways can be observed as the output (218). This approach will allow proper patient stratification, because the discriminating responses will come from the set of activated pathways rather than clinical outcomes. Another potential application involves studying macrophage activation by combining different biological pathways relevant to different stimuli (e.g., IFNγ, LPS, or IL-4). The networks will reflect different dynamic states, which may help illuminate the underlying mechanisms behind the spectrum of macrophage inflammatory response.

By combining network medicine with machine learning and AI, disease endotyping of various CVDs has great potential. Methods such as LIONESS allow construction of sample-specific networks, which permit association of demographic and clinical characteristics with each sample network. When applied to CVD, we can construct gene correlation networks for different subjects in the dataset and compare changes in edge-weights between different groups (e.g., disease and control) to find differentially changed interactions. While these networks differ from reticulotypes, which are genotype driven, sample-specific networks can aid precision medicine approaches in CVD.

Additionally, CVD research may benefit from integrative and diverse omics types in network models. Implementing methods such as Multi-omics Factor Analysis (MOFA) (219) and Similarity Network Fusion (SNF) (41) can illuminate molecular mechanisms underpinning distinct CVDs. In the context of gene regulation, combining the chromatin architecture through assays such as DNase-sequencing, ATAC-seq, or other epigenetic marks (e.g., DNA methylation) using SPIDER (201) to construct phenotype-specific gene regulatory networks can yield important regulators of disease biology. Enhancers play a crucial role in gene regulation and often act as an intermediary between genotype and phenotype relations; however, finding the genes they regulate is very challenging. Using GRN models with information about 3D organization of the genome (e.g., Hi-C) to find topologically associated domains combined with histone modifications on the DNA will allow us to identify not only enhancers in different cell types, but also the genes they regulate.

Combining and extending methods like PANDA and PUMA can explore the regulatory relationship between mRNA–miRNA–lncRNA by modeling different mechanisms of action. Such multi-dimensional networks will facilitate new therapeutic strategy for CVD and help understand its development.

Along with the number of technologies that identify diverse omics types, considerable success has been achieved at the cellular level in terms of these measurements. By allowing both hypothesis-driven and unbiased screening of newer cell types in the population, single-cell RNA sequencing technology has become almost a mainstay in various experimental explorations (220). Similarly, single-cell ATAC-sequencing and single-cell multiomics platforms with simultaneous ATAC-seq and gene expression can help construct regulatory networks for different cell states or types, allowing concomitant inference of regulatory landscape behind the expression profiles. In the context of CVD, a large amount of single cell multiomics data has already been generated. New network methods to study gene regulation using single cells will provide a fine perspective on various cell types and cell states affected by disease pathologies.

One of the most promising outcomes of the growing application of network medicine approaches in various diseases, including CVD, is the impetus to target discovery and well-designed clinical trials. Network medicine, along with machine learning-based approaches, provides a solid framework for identifying disease mediator biomolecules as drug targets. Moreover, the network view of drug development, which incorporates topological and functional modules of disease subnetworks, will allow repurposing already-approved drugs by finding alternate targets.

## Closing Thoughts

Despite its roots in graph theory and social networks, use of a network framework in biology formally began around the same time as publication of a draft human genome. In principle, the last 20 years of scientific progress in a variety of technological breakthroughs in data generation have outpaced the development of analytical frameworks required to process the vastness and depth of biomedical big data. Network medicine derives its methodological advancements from innovations in the field of social network analysis and complex network theory in physics. This vast area involves different facets and different types of networks, some elaborated in this review. Often, networks are (mis)used as merely a visualization tool to demonstrate differentially expressed genes or proteins and display functional enrichment analysis results; in other cases, we use complicated mathematical structure to extract information from a network, thus making it an esoteric construct restricted to specialists. Both problems may have limited the allure of network medicine. With increasing interdisciplinarity of scientists in cardiovascular research, network medicine is well poised to study the complexity, ontology, and epistemology of biomolecules in CVD context and to provide translational benefits.

## Author Contributions

AS wrote the initial draft, which was reviewed, edited, and revised by all authors. All authors listed have made a substantial, direct, and intellectual contribution to the work, and approved it for publication.

## Conflict of Interest

The authors declare that the research was conducted in the absence of any commercial or financial relationships that could be construed as a potential conflict of interest.

## Publisher’s Note

All claims expressed in this article are solely those of the authors and do not necessarily represent those of their affiliated organizations, or those of the publisher, the editors and the reviewers. Any product that may be evaluated in this article, or claim that may be made by its manufacturer, is not guaranteed or endorsed by the publisher.
